# Impact of phase II trials with progression-free survival as end-points on survival-based phase III studies in patients with anaplastic gliomas

**DOI:** 10.1186/1471-2407-7-106

**Published:** 2007-06-22

**Authors:** Victor A Levin, Sandra Ictech, Kenneth R Hess

**Affiliations:** 1 Department of Neuro-Oncology, Unit 431, The University of Texas M. D. Anderson Cancer Center, 1515 Holcombe Boulevard, Houston, Texas, 77030, USA; 2 Department of Biostatistics & Applied Math, Unit 447, The University of Texas M. D. Anderson Cancer Center, 1515 Holcombe Boulevard, Houston, Texas, 77030, USA

## Abstract

**Background:**

To assess progression-free survival (PFS) as the appropriate end-point for phase II trials for anaplastic gliomas (AGs) and to determine the impact of PFS on survival-based phase III trials.

**Methods:**

Combined data from 16 phase II studies (N = 529 patients) were analyzed to determine progression-free survival (PFS) at 6, 9, and 12 months and the impact of age, Karnofsky performance score (KPS), number of prior chemotherapies, and response to treatment on PFS.

**Results:**

The specific chemotherapy used was the major effector of PFS at 6, 9, and 12 months. Age, KPS, treatment response rate, and number of prior chemotherapies did not affect PFS to the same extent. Hierarchical cluster analyses and linear least squares fitting of PFS_9 _*v *PFS_12 _demonstrated the existence of three therapeutic efficacy groups with PFS rates at 6, 9, and 12 months ranging from lowest (A) to highest (C). The PFS_6 _was 15% in group A and 41% in group C (p < .0001); the PFS_12 _was 9% in group A and 33% in group C (p < .0001). Further, 80% of patients at recurrence had a 23% likelihood that each chemotherapy would provide > 1 year of additional life.

**Conclusion:**

Based on PFS rates at 6, 9, and 12 months for AG patients, a differential of 1.5 to 2 years is the norm and could invalidate overall survival as an end-point for phase III studies in patients with AG. PFS is a more reliable end-point because it reflects the true antitumor benefit of the chemotherapy.

## Background

Anaplastic gliomas (AGs) constitute a group of WHO grade III primary brain tumors that include anaplastic astrocytoma (AA), anaplastic oligodendroglioma, [1] anaplastic mixed oligoastrocytoma (AOA), and anaplastic ependymoma. [2-4] As a group, the incidence of these tumors is approximately 1.6/100,000, which translates into about 4400 patients who are diagnosed with the tumor yearly in the U.S. [5,6]

AGs typically infiltrate (invade) adjacent brain. However, in patients with the different types of AG, chemotherapy agents show a discrepant ability to traverse the normal vasculature as well as tumor capillary beds and to achieve therapeutic levels in and surrounding infiltrating tumor cells. To date, most agents considered "active" in patients with these tumors are lipophilic alkylating agents such as BCNU (carmustine), CCNU (lomustine), procarbazine, and temozolomide. [7-21] While there has been much progress in the treatment of AG over the years, with the median survival time seen just a few years ago going from 13 to 19 months in patients treated with surgery and irradiation [22,23] to 6.5 years in patients in a recent randomized trial of alpha-difluoromethylornithine (DFMO, eflornithine) plus procarbazine, CCNU, and vincristine (PCV) *v *PCV, [24] curative treatments with reduced regional and systemic toxicity are still very much needed.

However, one of the hindrances to the introduction of new agents for AGs, and in particular AAs and AOAs, into clinical use is that many of the phase II studies of single-agent therapy and drug combinations in patients with AAs and AOAs have shown a considerable disconnect between the percentage of patients whose disease responds to or stabilizes (i.e., does not progress) in response to treatment and the durability of the treatment response, defined as the time-to-tumor progression (TTP) or as progression-free survival (PFS). For example, complete response (CR), partial response (PR), minor response, and stable disease rates of 0% to 83% have been reported, with a median time to tumor progression (MTP) of 6 to 49 weeks and with only a modest correlation between the rates of response, stable disease, and MTP. [18,25-37] Thus, it is difficult to draw firm conclusions from these studies with regard to the effectiveness of the treatment under study. On the basis of these and other observations, we believe that the PFS rate at a specified time point between 6 and 12 months provides a realistic time frame for trials evaluating agents for AG. That is, the use of PFS as the primary end-point in phase II trials has the advantage of enabling trials to be shorter in duration, plus PFS is a practical and achievable surrogate indicator of actual long-term prognosis. PFS rates at 6, 9, and 12 months (PFS_6_, PFS_9_, and PFS_12_) are also good end-points, both for drugs that cause tumor shrinkage and for those that just delay TTP.

We will demonstrate that PFS is a more realistic and accurate end-point than overall survival (OS) for chemotherapy trials of today's new agents in patients with mid- and high-grade gliomas. [24] In this report, we will also describe the value of PFS_6_, PFS_9_, and PFS_12 _and how these end-points might be applied so that the clinical development of effective new drugs and drug combinations can be accelerated.

## Methods

To better define whether there would be any benefit to studying PFS_6_, PFS_9_, or PFS_12 _and to evaluate the impact of age, Karnofsky performance score (KPS), and number of prior chemotherapies on PFS, covariates typically associated with survival outcome, we collated a large retrospective database of patients with AA or AOA treated on phase II studies. Patients with anaplastic ependymoma were excluded because of the relative rarity of this tumor. Patients with AOs were also excluded because these tumors are relatively more sensitive to chemotherapy and radiation therapy, and thus these patients should be analyzed separately from those with AA or AOA.

Data in the database were obtained from the following sources: the same patient database used in an earlier study [38]; reports of single-agent therapy with imatinib (Gleevec) [39] or temozolomide [21,40]; portions of randomized trials of interferon-beta *v *interferon-beta with isotretinoin [38,41], carboplatin and isotretinoin [42], and carboplatin, 5-fluorouracil, and procarbazine [43]; a trial of DFMO performed at The University of Texas M. D. Anderson Cancer Center (UTMDACC) and the University of California San Francisco [37]; a published trial of temozolomide and marimastat [44], and the institutional database for a number of additional trials conducted at UTMDACC and generated by Data Management Services at UTMDACC from 1999 through 2004 [45]. The UTMDACC database consists of data from sequential phase II studies. Some study findings were published only in abstracts and others were from cooperative group studies. In all cases, the computer database was available for interrogation for these analyses. Aside from the number of previous chemotherapies, these studies had common eligibility criteria, and one of us [39] was an investigator in each study. The latter dataset was verified, to the extent possible, by (a) requiring that the tumor pathology be reviewed prior to the study by a UTMDACC neuropathologist; (b) using common MRI criteria for the determination of response and tumor progression [46]; (c) removing patients from the analysis with biopsy and/or strong radiological evidence of radiation necrosis [47-49]; (d) including only patients treated on phase II studies whose full prior treatments could be fully documented; (e) including only patients annotated evaluable in the database; and (f) including only patients for whom full follow up information was available. The entry requirements for all studies were also similar: KPSs of ≥ 60 at study entry, unequivocal radiographic evidence of recurrence (progression), age ≥ 16 years, absolute neutrophil count ≥ 1500, platelet count ≥ 125,000, and results of chemistry and liver function tests within 1.5 to 2.0 times the normal value, depending on the protocol. All patients had signed an IRB-approved consent form agreeing to a formal protocol treatment and/or the use of their computer-based record.

## Results

Data on patients from 16 phase II studies conducted between 1995 and 2004 were collected. A total of 529 patients were included in the database for analysis. For all studies and the MDACC database, complete and partial response were determined based on the brain MRI using the Macdonald criteria [46]. Comparison of the 16 therapy groups showed no differences among the groups in terms of age and the KPS (Table 1). Table 2 summarizes the mean, SEM, and range for the mean age, KPS, response (CR+PR) rates, and number of prior chemotherapy treatments for each treatment group from Table 1. In addition, t-tests for each covariate found no differences among the 16 protocols from the standpoint of patient age, KPS, treatment response, and number of prior chemotherapies.

**Table 1:** Age, KPS, CR/PR, PFS, and prior chemotherapy regimen demographics of studies^a^

**Trial No.**	**Drugs**	**N**	**PR/CR**	**PFS_6_**	**PFS_9_**	**PFS_12_**	**Age (SD)**	**KPS (SD)**	**No. Prior Chemo (SD)**
1	Gleevec, phase I/II [39]	22	0%	7.7%	4.8%	3.4%	46.8 (11.5)	83.2 (10.9)	1.3 (0.5)
2	DM86-15: IV carboplatin [38]	11	9%	24.9%	18.6%	6.5%	36.3 (8.6)	82.7 (10.1)	1.0 (0.0)
3	Miscellaneous agents^b ^[45]	32	0%	14.2%	9.8%	9.3%	40.5 (12.4)	84.2 (14.2)	2.7 (0.6)
4	Carboplatin with VP16 [42, 45]	19	5%	11.9%	10.4%	9.5%	41.3 (8.9)	85.4 (13.9)	3.0 (0.8)
5	CPT-11 alone or with thalidomide, CDDP, or tamoxifen [45]	22	9%	21.0%	15.0%	15.0%	41.3 (10.2)	80.0 (13.6)	3.0 (0.9)
6	DM92-109: interferon-beta arm [38, 41]	28	17%	20.5%	18.4%	16.7%	42.7 (9.4)	85.4 (8.8)	1.5 (1.0)
7	DM88-130: DFMO [37]	42	14%	29.1%	21.1%	19.1%	38.0 (11.2)	84.3 (11.7)	1.8 (1.2)
8	Temozolomide [40]	126	33%	46.0%	25.0%	19.6%	42.6 (11.2)	83.9 (10.8)	1.1 (0.8)
9	Temozolomide with cRA [45]	37	11%	38.6%	23.8%	20.8%	41.0 (11.4)	84.3 (14.8)	2.4 (0.7)
10	DM92-109: interferon-beta with cRA arm [38, 41]	30	7%	42.0%	28.9%	22.0%	42.5 (8.3)	84.6 (8.1)	1.5 (0.9)
11	Temozolomide [45]	56	4%	33.2%	29.3%	23.3%	39.4 (9.5)	84.6 (11.4)	2.5 (0.6)
12	cRA [45]	27	11%	29.2%	25.9%	25.9%	42.2 (12.8)	83.0 (13.0)	2.6 (0.8)
13	BCNU or CCNU alone or with 6-TG [45]	31	13%	36.4%	36.4%	27.5%	44.1 (14.4)	83.5 (11.9)	2.3 (0.5)
14	Carboplatin with cRA [45]	15	0%	44.4%	36.8%	31.5%	39.1 (9.5)	82.9 (13.8)	2.7 (0.8)
15	Temozolomide with marimastat [44]	18	7%	40.0%	38.2%	36.4%	38.6 (9.9)	88.2 (13.3)	2.2 (0.4)
16	DM89-092: carboplatin with 5-FU & procarbazine [38, 43]	13	17%	47.1%	43.9%	41.1%	43.5 (16.9.9)	81.7 (11.9)	0.9 (1.0)

^a^Data are ordered by ascending PFS_12 _values.

^b ^Thalidomide, Iressa, CI-980, etoposide, tamoxifen, Gleevec, R115577, fenretinide, topotecan, menogaril

^c ^Only median values cited in reference.

Abbreviations: Chemo, chemotherapies; KPS, Karnofsky performance score; CR, complete response, PR, partial response; PFS, progression-free survival; SD, standard deviation; VP16, etoposide; CPT-11, irinotecan; CDDP, cisplatin; cRA, isotretinoin or 13-cis-retinoic acid; 5-FU, 5-fluorouracil

**Table 2:** The mean, SEM, and range of ages, KPS, response (PR/CR), and number of prior chemotherapies for the 16 groups*

	**Age**	**KPS**	**PR+CR**	**Number of prior chemotherapies**			
				
				**0**	**1**	**2**	**≥ 3**
**Mean**	41	84	10%	2.5%	52%	34%	9%
**SEM**	0.7	0.5	2.2	1.8	6.0	4.7	2.3
**Group ranges**	36–47	80–88	0–33	0–24	10–100	0–69	0–26

*T-test comparisons for all variables among the 16 groups were not significantly different. KPS = Karnofsky performance score; PR = partial response; CR = complete response.

**Figure 1:**
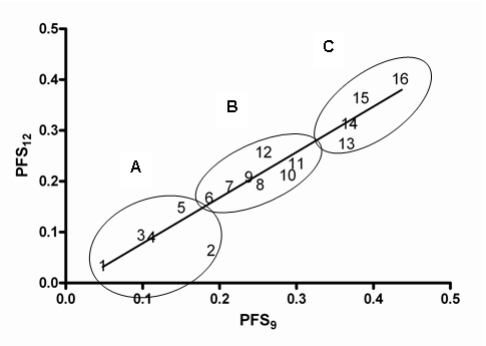
Linear regression plots of the treatment pairs PFS_9 _versus PFS_12_. Circled areas A, B, and C are regions coincidental with the clusters yielded by the hierarchical cluster analysis.

To better discriminate the PFS data in Table 1, we plotted PFS_6 _*v *PFS_12_, PFS_6 _*v *PFS_9_, and PFS_9 _*v *PFS_12 _to determine goodness of linear fit. The worst fit to the linear model was seen for PFS_6 _*v *PFS_12 _(r^2 ^= .64), and the best fit to the linear model was seen for PFS_9 _*v *PFS_12 _(Fig. 1; r^2 ^= .90). We then analyzed the PFS data at 6, 9, and 12 months together with age and KPS using hierarchical cluster analyses with complete linkage and Euclidean distances (Statistica for Windows, version 5.5). This analysis yielded three groups, which we designated A, B, and C, shown graphically in Figure 1. The respective protocol groups, the number of patients in the groups, and the respective weighted mean PFS_6_, PFS_9_, and PFS_12 _for groups A through C are summarized in Table 2. Of the 529 patients in the dataset, 20% were in Group A, 65% were in Group B, and 15% were in Group C.

**Figure 2:**
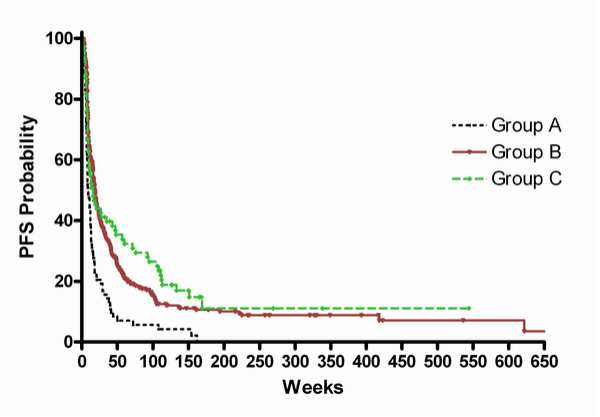
This is the Kaplan-Meier plot of the probability of PFS for patients from Groups A, B, and C.

From Table 3 and Figures 1 and 2, it appears that Group A is markedly inferior to groups B and C in terms of PFS. To test the statistical significance of this observation, we performed a Cox regression analysis using covariates of age, prior chemotherapy, and treatment group. KPS could not be used as a covariate in this analysis since time- and patient-coupled KPS data were incomplete. This analysis, the results of which are summarized in Table 4, showed that age at treatment was a significant factor (p = .006) but that membership in treatment groups B and C was a more significant factor, with treatment groups B and C superior to group A from the standpoint of PFS (p < .0001).

**Table 3:** Weighted PFS values for the three clusters (groups)*

**Groups**	**Trial No.**	**N**	**PFS_6_**	**PFS_9_**	**PFS_12_**
**A**	1–5	106	15%	11%	9%
**B**	6–12	346	37%	25%	21%
**C**	13–16	77	41%	38%	33%
**B+C**	6–16	423	38%	27%	23%

* Groups are shown in Figure 1, and the values were calculated from data in trials summarized in Table 1.

PFS for groups B+C are weighted means of averages shown for groups B and C.

**Table 4:** Results of Cox regression analysis*

**Covariate**	Hazard function** (95% confidence intervals)	**p-value**
**Age**	1.01 (1.00, 1.02)	.006
**Prior chemotherapy**	1.02 (0.92, 1.14)	.659
**Group B**	0.48 (0.35, 0.66)	<.0001
**Group C**	0.51 (0.40, 0.65)	<.0001

*Analysis was performed using treatment groups A through C as a categorical indicator and Group A as the reference.

**Hazard function if less than 1 it indicates increased PFS and, if greater than 1 decreased PFS. In the case above, Groups B+C are associated with statistically increased PFS.

## Discussion

What are the implications of this study for phase II and phase III chemotherapy trials? At one level, we wanted to define what represented a "good" outcome from the standpoint of PFS_9 _and PFS_12_. To that end, we were able to define an indicator of efficacy in a phase II trial in AG patients; we found that a therapy that produces a PFS_6 _of ≤15%, regardless of the number of prior chemotherapies, is not likely to be worth pursuing further. Of the remaining 80% of patients who made up groups B and C, PFS_6 _was similar for the two groups (37% vs. 41%); the PFS_9 _and PFS_12 _for the two groups, however, differed by 13% (25% vs. 38%) and 12% 21% vs. 33%). The difference between PFS_9_and PFS_12 _was only 4% for Group B and only 5% for Group C. Thus, for all practical purposes, we believe clinical trial investigators can easily set the PFS bar at 6 months or 9 months and be confident that this will distinguish inferior from adequate trial outcomes based on the values in Table 3.

From our own clinical experience and the experience of investigators in clinical phase II trials published over the years, the value of treatment response in determining the value of a new therapy for recurrent AGs remains problematic. Our current study supports that position, in that the use of the overall response rate, the historical phase II criterion, would have inadequately predicted the true benefit and limitations of many of the therapies analyzed in this study. This disconnect between the response rate and the durability of response (see Tables 1 and 2 and Fig. 1) is not a new concern for those conducting clinical trials in patients with high-grade gliomas. One reason for this disconnect is undoubtedly the limited antitumor activity of today's chemotherapy agents. Another reason proposed in the past is that the central nervous system does not rapidly remove dead glioma tumor cells. [50] Thus, the time before tumor cell kill can be visualized may be considerably delayed and lost in the process of a clinical follow up, unless meticulously sought. [51] This makes it difficult to pinpoint the actual rate or durability of response.

Furthermore, there is a mistaken belief that determining response is easier than determining tumor progression in patients with AG, but also that response is the only true indicator of drug effect for phase II trials. Historically, however, determining a response has frequently been more difficult than determining tumor progression (treatment failure). [49] This becomes even more of a problem, however, when pharmaceutical companies insist that patients not be entered into phase II trials until their initial therapy has failed, which for patients with AG could be the time it takes to develop postoperative gliosis or a radiation effect. Such a practice has the potential to lead to a disturbing false-positive response rate because the effectiveness of treatment is based on the resolution of postoperative changes and not the effects of treatment. While the same could be said for defining tumor progression in the face of radiation effects, the definition of therapeutic failure appears to be more reliable. [46,52] Hence, it is the current belief of many clinical trial neuro-oncologists that PFS is a much better indicator of treatment efficacy than the response rate. Certainly it is our hope that future therapies will be so effective that complete responses will be seen regularly and the therapeutic bar will be raised even higher, but until that time, it is important to consider PFS a legitimate measure for studies of single agents and drug combinations for the purpose of community usage but also, in some cases, for marketing approval.

How treatment-defining phase III clinical chemotherapy trials for patients with malignant gliomas are designed and conducted is a concern. Currently, OS is considered the benchmark for assessing the benefit of new chemotherapy, radiotherapy, or chemoradiation regimens in phase III trials conducted in patients with glioblastomas (WHO grade 4). This end-point is reasonable and acceptable in this setting at this time, since, for the most part, the Kaplan-Meier probability of surviving declines exponentially and survival in patients treated with current agents minimally deviates from this expectation. In phase III trials in patients with AG, however, OS is arguably the wrong study end-point. This is because, as shown in Table 3, there are a number of chemotherapy treatments that can significantly prolong life after first tumor progression and thereby lengthen OS, independently of the phase III trial regimen being tested. For example, by extrapolating from Table 3, one could predict that 80% of patients in our phase II studies have, on average, a 23% (21% to 33%) chance of living more than 1 year for each treatment protocol they receive. How, then, might this impact the interpretation of a recent randomized phase III study of an effective agent?

To answer this question, we examined our findings from the current study of PFS in phase II studies in light of the results from the recent phase III randomized trial of DFMO-PCV *v *PCV alone for patients with AG tumors. [24] In that study, there was a marked difference in the median PFS between the two treatment arms, but there was far less of a difference in the median OS between the two treatment arms. [24] In particular, the median PFS was 71 months and the median OS was 76 months in the DFMO-PCV group, but the median PFS was only 38 months while the median OS was 61 months in the PCV group. We reviewed this study in May 2006 to determine how many patients had had disease progression and what treatment options they had then elected. We found that 46% of the DFMO-PCV patients and 41% of the PCV patients had been censored during the 13 years since study inception. Furthermore, among non-censored patients, we found that 34% of patients received an average of 1.6 chemotherapies after tumor progression, 22% underwent 1.1 further surgeries, 3% underwent 1.0 re-irradiation, and 15% of patients were treated with more than one modality. Thus, no matter how careful the randomization, one could not account prospectively for the fact that one group required more therapies at recurrence than did the other group. The fact that a number of contemporary phase II trials of chemotherapies for AGs produce substantial palliation (21% to 33% PFS_12_) means that OS as an end-point would not truly reflect the benefit of the primary phase III regimen. In the case of the DFMO-PCV trial discussed above, only 23 months (OS – PFS) of successful phase II chemotherapies would have been needed. In addition, in the cited example above, reoperation and repeat irradiation were also used after tumor progression.

For these reasons, we strongly question whether phase III studies with OS as the primary end-point can or should be conducted in patients with AGs at this time. We argue that OS as an end-point for phase III AG studies today is a flawed objective and that its use will result in new and effective regimens tested in phase III trials being judged ineffective (false negative) for the treatment of AG.

## Conclusion

We conclude from our analysis that PFS is a much more valuable and reliable end-point for phase III studies than overall survival, because it reflects the true antitumor benefit of the chemotherapy being studied. Since the median survival of AG patients can range from 4 to 6 years, focusing on PFS in phase III studies would also allow studies to be performed and concluded in possibly half that time. It is hoped that this article will help to convince clinical trial investigators, the pharmaceutical industry, and government regulators to develop robust strategies that utilize PFS as the primary end-point in trials of much-needed therapies for patients with AGs.

## Competing interests

The author(s) declare that they have no competing interests.

## Authors' contributions

VAL conceived of the study, performed some of the statistical analyses, and wrote the paper. SI collated the datasets and verified the MDACC internal database to the extent that patients or their families were contacted for long-tem follow up status. KRH proposed statistical approaches and computed some of the analyses and provided editorial comments regarding their interpretation and figure presentations. All authors read and approved the final manuscript.

## Pre-publication history

The pre-publication history for this paper can be accessed here:


